# ESG Investment Scale Allocation of China’s Power Grid Company Using System Dynamics Simulation Modeling

**DOI:** 10.3390/ijerph20043643

**Published:** 2023-02-18

**Authors:** Birong Huang, Zilong Wang, Yuan Gu

**Affiliations:** 1 College of Economics and Management, Nanjing University of Aeronautics and Astronautics, Nanjing 211106, China; 2 School of Electrical Engineering, Southeast University, Nanjing 210096, China

**Keywords:** Power Grid Company, SD, ESG investment, scale allocation

## Abstract

In recent years, with the global recognition of the concept of sustainable development, the international market attaches great importance to the Environment, Society, and Governance (ESG) investment performance of enterprises. The “carbon peaking and carbon neutrality” goal puts forward requirements for Chinese enterprises to carry out ESG investment. As a large state-owned enterprise in China, power grid companies need to take the lead in ESG investment. Based on the System Dynamics (SD) theory, this paper establishes the simulation model of ESG-responsible investment of power grid companies, including the environmental investment sub-module, social investment sub-module, and governance investment sub-module. Taking a provincial Power Grid Company as an example, the numerical simulation of ESG investment of power grid companies is carried out. The actual input-output efficiency of ESG investment of power grid companies is reflected through the mapping relationship between key indicators and investment amount, and the ESG investment scale and investment weight of the Power Company in the coming years are predicted. Compared with the traditional static analysis method, this model can provide a theoretical basis for power grid companies to carry out ESG investment decisions.

## 1. Introduction

Since the 21st century, the problem of environmental resource scarcity and global warming has become increasingly serious. In order to pursue long-term sustainable investment returns, the enterprise investment concept with ESG as the core emerged at a historic moment [1]. Many scholars have studied the relationship between ESG investment and the sustainable development of enterprises and confirmed that the improvement of enterprises’ operating efficiency depends on their ESG performance [2,3,4,5]. After decades of research and development, ESG investment has become the mainstream of investment activities in the global capital market [6,7].

Focusing on China’s sustainable development in the context of globalization, in order to reduce carbon emissions, the proposed goal of “carbon peaking and carbon neutrality” provides a deep soil for the development of ESG investment in China’s power grid enterprises. References [8,9] studied the impact of enterprise operations on energy efficiency and regional carbon neutrality. Reference [8] points out that the expansion of urban commercial banks improves energy efficiency by easing financing constraints, which is conducive to achieving regional carbon neutrality in China. Reference [9] studied the role of green technology innovation of listed companies in achieving carbon-neutral development of cities and pointed out that green technology progress of enterprises can effectively improve energy efficiency and promote carbon-neutral development. Zhu et al. (2019) studied the relationship between the strictness of environmental policies and the efficiency of regional power production and pointed out that the change in power production technology has significantly affected the development of regional environmental policies and a low-carbon economy [10].

Nowadays, many algorithms have been applied to the simulation and analysis of power systems. For example, the literature [11] proposed a new two-stage hybrid domain decomposition algorithm, which can simulate various kinds of interference and solve a large number of emerging problems such as prospective dynamic simulation of power systems, real-time operation enhancement, and dynamic security evaluation. In the study [12], a mixed domain decomposition algorithm based on Schwarz-Schur complement was used to simulate power electronic dominated grids, thus speeding up the dynamic simulation and analysis of power systems. The above algorithms are mostly used in the field of dynamic and stable operation of power systems, and more effective algorithms need to be explored for the sustainable development management and investment benefit evaluation of power enterprises. To study the sustainable development benefits of enterprises and guide the investment decisions of enterprises, the literature [13] uses the dynamic system method to design the investment model and develop the irrigation system to realize the sustainable management of water resources, which is helpful to improve the environmental benefits in the ESG indicators of enterprises. In the literature [14], a genetic neural network model was developed using a genetic algorithm with global optimization, which has a good predictive power for the financial distress of listed companies and has a great advantage in predicting the economic benefits of corporate ESG performance.

This paper introduces SD theory to carry out ESG investment scale allocation of power grid companies. SD modeling theory was first proposed by Forrester [15,16], which is a typical method for studying system structure and behavior and can understand the behavior patterns of the system at different stages over time. Based on the causal logic relationship between various factors, SD can quantify abstract and general systems concretely and is often used to solve nonlinear and complex dynamic system problems [17]. Thus far, SD has been applied in many fields [18,19,20,21]. Wen and Bai (2017) built an SD model to simulate the impact of different strategies on urban traffic carbon emissions [18]. Nassery et al. (2017) established an SD model for water management in semiarid areas [19]. Xiu et al. (2019) applied SD to support the production and operation decisions of metal mines [20]. Zhang (2022) applied SD to explore the causes of enterprise financial risk and the conduction path of risk sources [21]. SD has also been applied in power system-related fields. Based on SD, the literature [22] constructs a deduction model for the development of power system supply-side morphology under the dual-carbon target. The literature [23] studied the impact of transmission and distribution electricity price policy on the cash flow of power grid enterprises and established an enterprise investment optimization decision-making model using SD theory.

In fact, the existing SD model often only analyzes the investment benefits of electric power enterprises in a certain aspect of ESG. There is nothing in the literature to evaluate and analyze the comprehensive benefits of ESG investment in electric power enterprises. On the basis of the existing literature, this paper uses the SD model to study the scale allocation of ESG investment in electric power systems to improve the comprehensive benefits of ESG investment in electric power enterprises. The advantages of using SD to guide ESG investment scale allocation of power grid companies are mainly reflected in:

- In essence, the process of power grid investment scale allocation is a complex system process with dynamic feedback;
- With the rapid development of the economy and society, the investment objectives and investment capabilities of power grid companies change dynamically over time;
- The actual system of power grid investment benefits includes a variety of influence variables, among which there is a complex nonlinear relationship, so the characteristics of the system are consistent with the characteristics of the SD itself.

This paper first defines the ESG investment system of the power grid into the environmental subsystem, the social subsystem, and the governance subsystem, and analyzed its internal causality. Then, the functional relationship between variables is established, and the SD model is established. Finally, taking the investment and construction of a provincial power grid in China as an example, based on the SD theory, the mapping relationship between the power grid investment and indicators in this region is simulated, and the investment scale guidance of the power grid companies in this region and the investment weight of each indicator is given in the coming years.

The main contributions of this paper are:

- In this paper, the ESG investment benefits of electric power enterprises are comprehensively evaluated, rather than only studying the environmental, social, and economic benefits of enterprises.
- Compared with the traditional static analysis method, the SD model used in this paper can analyze the ESG investment benefits of power enterprises on multiple time scales, which is helpful to guide the investment decisions of power enterprises dynamically.
- This paper uses SD modeling and practical mathematical calculation to give the ESG investment scale allocation plan for power enterprises in the next three years so that the investment decision of power enterprises has sufficient data support.

Our article is structured as follows: Section 2 is our method description. Section 3 describes our empirical analysis and results discussion. Finally, Section 4 provides the conclusions.

## 2. Methods

### 2.1. System Description

The ESG investment system of the company is a dynamic system in which ESG factors interact and coordinate with each other [24]. No matter which factors are out of balance, it may lead to the loss of investment benefits. The ESG investment system of a power grid company examines the relationship between the investment amount of a power grid company in ESG and the improvement of investment efficiency. The improvement of ESG efficiency is reflected by the changes in key impact indicators. ESG investment scale allocation of power grid company based on SD including the following steps:

1. From the three aspects of ESG, select the key evaluation indicators that can represent the investment benefits;
2. Analyze the causality between indicator variables and design a causality diagram;
3. Build the SD simulation model. Determine the state variables, rate variables, and auxiliary variables in the simulation model, as well as the functional relationship equations among the variables;
4. Use the historical data to simulate the output results of the system under different conditions, and the model parameters are adjusted based on the comparison between the model output value and the actual value until the model meets the validity test;
5. The adjusted model is used to simulate and predict the future ESG investment scale and investment weight of power grid companies under the given expected values of ESG indicators.

### 2.2. System Causality Analysis

SD focuses on the dynamic feedback changes of a system under the combined influence of internal and external factors and looks at the internal structure of the system to find the root cause of the problem, rather than using external disturbances or random events to explain the system’s behavior, enabling researchers to conduct policy recommendations.

**Figure 1:**
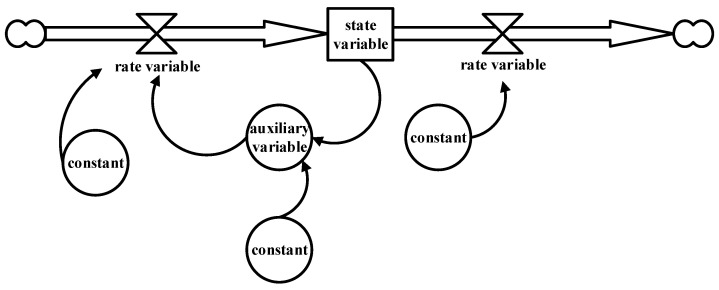
The basic structure of SD.

First-order feedback loops are the basic structures that make up a system, as shown in Figure 1. First-order feedback loops are loops that couple the state variables, rate variables, auxiliary variables, and constants of the system, and complex systems consist of these interacting feedback loops.

**Figure 2:**
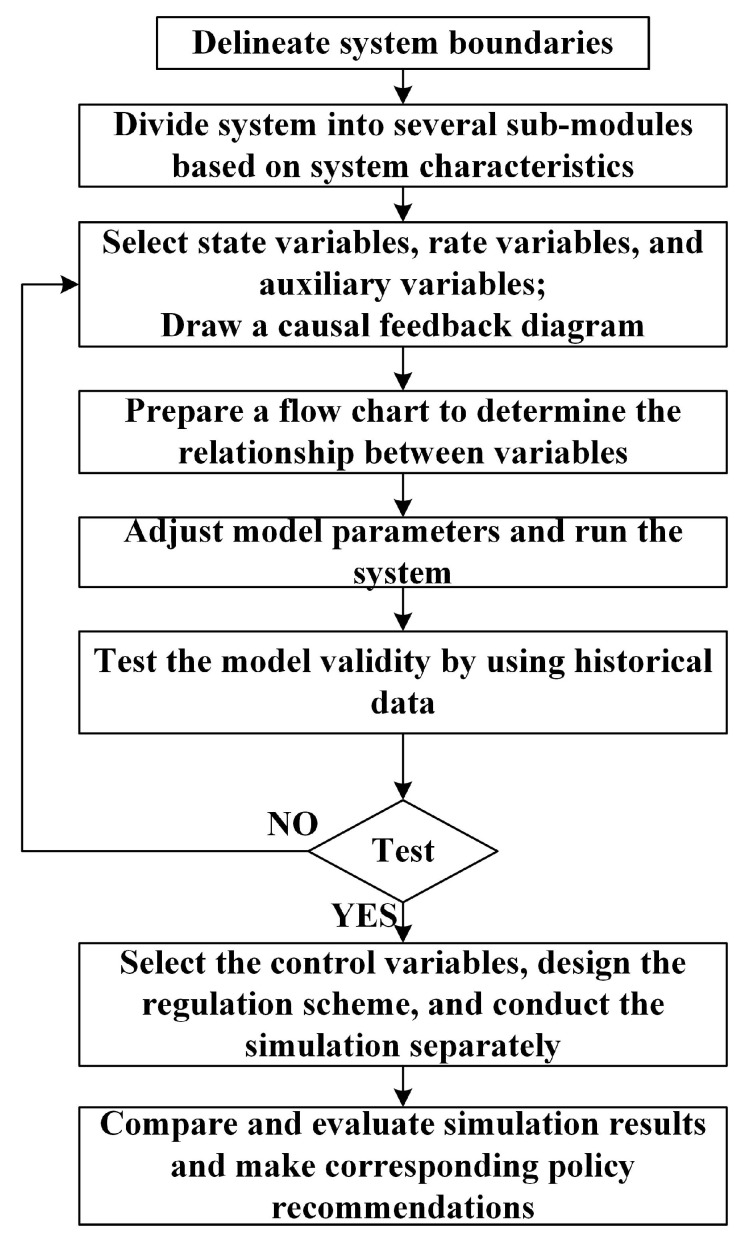
SD flow chart.

To solve the problem with SD, first, define the problem and delineate the system boundary. Then define the types of variables and rationalize the variable causality, conceptualize the system flow diagram and the feedback coupling relationship of each loop. Finally, abstract the corresponding simulation model, transform the qualitative description into quantitative analysis, and show the logical relationship between variables through images to make the research results clearer and more understandable. The flow chart is shown in Figure 2.

The focus of SD is to establish the coupled feedback relationships between its internal variables, which are complex and mostly dynamic. In the case of power grid investment construction, the process is not static, but essentially a complex system process with dynamic feedback, where multiple influencing factors intertwine and balance each other. Eventually quantitatively modeling the benefits of power grid investment in the future development within a certain time scale, including ESG benefits, to achieve the purpose of dynamic assessment.

In this section, the investment system of power grid companies is divided into an environmental subsystem, social subsystem, and governance subsystem, and the interaction of indicator variables in each system is explored to build a causality diagram of the whole and each subsystem, where “+” represents positive causality, and “−” represents negative causality.

#### 2.2.1. Causality Analysis of Environmental Investment Subsystem

**Figure 3:**
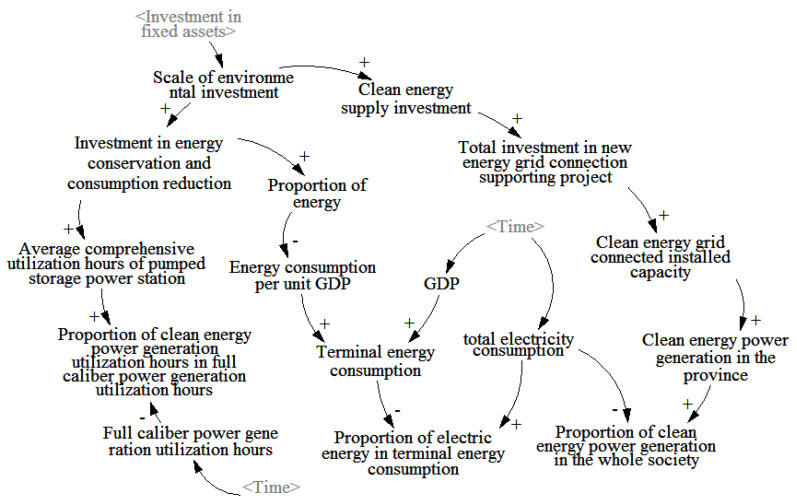
Cause and effect analysis diagram of environmental investment subsystem of grid company.

The environmental investment of the power grid company focuses on the strategic goal of “carbon reaching the peak and carbon neutralization”, which is expressed by the mapping relationship between the investment and construction of the power grid company and the development of renewable energy utilization. The main indicators of the causality loop of the power grid investment environment subsystem studied by the project include the average comprehensive utilization hours of pumped storage power plants, the energy consumption level per unit GDP, the electrical energy in the terminal energy, clean energy grid-connected installed capacity, the clean energy grid-connected installed capacity, the proportion of clean energy generation in the Province in the electricity consumption of the whole society, the proportion of energy-saving equipment, etc. The specific causality loop between these indicators and the power grid investment is shown in Figure 3.

#### 2.2.2. Causality Analysis of Social Investment Subsystem

The causality loop of the social investment subsystem of the grid company is mainly characterized by the correlation between the grid investment and construction in specific areas and the power consumption level of local users. The social investment and construction of the power grid should be able to continuously improve the power supply guaranteeing the ability and stability of the corresponding regions. In addition, based on the requirements of actively promoting the energy system revolution in China’s energy “four revolutions” policy, social investment in power grid construction should adhere to the market-oriented reform direction. On the other hand, China’s “14th Five Year Plan” outlines that it is necessary to accelerate the intelligent transformation of power grid infrastructure and the construction of smart microgrids, and improve the power system’s complementary and intelligent regulation capabilities.

**Figure 4:**
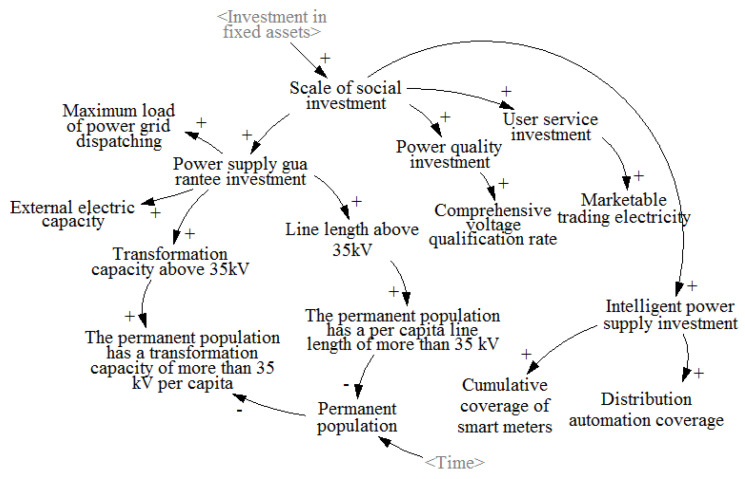
Cause and effect analysis diagram of social investment subsystem of grid company.

The causal loop between social investment and key indicators is shown in Figure 4. The main indicators of social benefits of power grid investment include the maximum load of power grid dispatching, external power capacity, line length above 35 kV per capita of the permanent population, transformation capacity above 35 kV per capita of the permanent population, comprehensive voltage qualification rate, market-based trading electricity, distribution automation coverage, and cumulative smart meter coverage.

#### 2.2.3. Causality Analysis of Social Investment Subsystem

**Figure 5:**
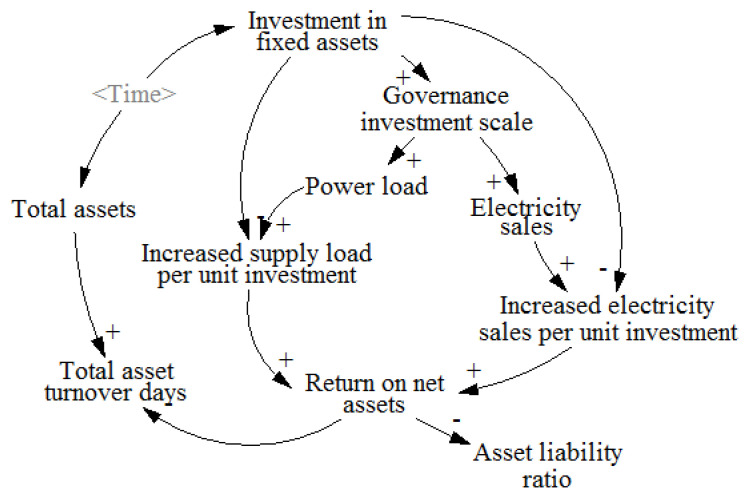
Cause and effect analysis diagram of governance investment subsystem of grid company.

The causality loop of the governance investment subsystem of power grid companies is mainly characterized by the relationship between the scale of power grid governance investment, electricity sales, and load growth. In recent years, the rapid development of China’s national economy has led to the continuous improvement of people’s consumption level, which is reflected in the growth of electricity load and electricity sales. The growth of unit investment power supply load and unit investment electricity sales can improve the net asset income of grid companies and reduce the asset-liability ratio. When the total assets are fixed, the increase in operating income also corresponds to the decrease in total asset turnover days. The specific causal loop of the governance investment of the grid company is shown in Figure 5. The main indicators of the governance benefit of the distribution network investment include the unit investment to increase the supply load, the unit investment to increase the sales of electricity, the return on equity, the total asset turnover days, the asset-liability ratio, etc.

### 2.3. SD Modeling

**Figure 6:**
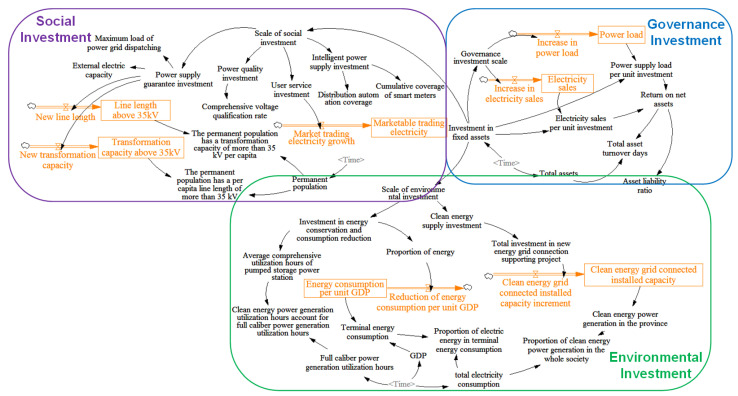
SD Model of ESG Investment of Power Grid Company.

According to the causal analysis of the responsible investment subsystem of the power grid company in the preceding chapters, the SD simulation model of the responsible investment of the power grid is designed as shown in Figure 6. The SD model mainly includes state variables, rate variables, auxiliary variables, and constants. The state variables are represented by box symbols in the model and are the final state of the system behavior; the rate variable is represented by the valve symbol in the model. Its main function is to influence the state variable and reflect the speed of its change. It is an intermediate variable from the state variable to the rate variable; auxiliary variables are used to assist calculation to obtain rate variables and state variables; a constant is a constant quantity. In this paper, seven variables are selected as the state variables, including the line length above 35 kV, market trading electricity, load, electricity sales, and clean energy grid-connected installed capacity. The rate variable selects seven variables, including the length of new lines, new transformation capacity, load growth, and sales growth. The auxiliary variables include 34 variables, such as average comprehensive utilization hours of pumped storage power plants, return on net assets, investment in fixed assets, and resident population. The SD model constructed in this paper does not contain constants.

## 3. Empirical Analysis and Results Discussion

### 3.1. System Parameter Setting

This paper simulates the responsible investment of a provincial Power Grid Corporation in China from 2016 to 2025, in which the historical data from 2016 to 2022 are used to test the validity of the model, and the responsible investment scale of the Power Grid Corporation from 2023 to 2025 is predicted. The data before 2016 in the company system are incomplete to meet the model calculation requirements, and the investment situation of power companies before 2016 is significantly different from the current one. The data from 2016 to 2022 can already guarantee the accuracy of model fitting.

The setting of model parameters is based on the actual situation of power grid development and construction in the Province, and some parameters are reasonably calculated according to the relevant development planning documents of the “Fourteenth Five Year Plan”, the expected data of authoritative institutions or the relevant foreign experience data. The initial values of model variables are shown in Table 1.

Then, according to the actual investment situation of the Power Grid Corporation and the relevant planning research reports of China’s “Fourteenth Five Year Plan”, the relevant auxiliary variables in the grid investment benefit simulation system established in this paper are given in the form of table functions, which are generally used to describe the nonlinear relationship between the two variables. If the nonlinear function relationship can be expressed in graphical form, it can be expressed in the form of DYNAMO table functions. The expression of the table function in SD is WITH LOOKUP (TY, X, XMIN, XMAX, XINCR), where TY is the table name, X is the argument, XMIN is the minimum value of X, XMAX is the maximum value of x, and XINCR is the value interval of x. The table functions of total assets, investment in fixed assets, GDP, power consumption of the whole society, utilization hours of full caliber power generation, and the number of permanent residents are shown in Table 2.

### 3.2. Model Validation

This paper tests the validity of the model with the relevant data on responsible investment of the Power Grid Corporation from 2016 to 2021. The model is used to simulate and calculate the data of the main variables from 2016 to 2021, and the actual values of the variables are compared with the simulation values to observe their fitting degree.

**Figure 7:**
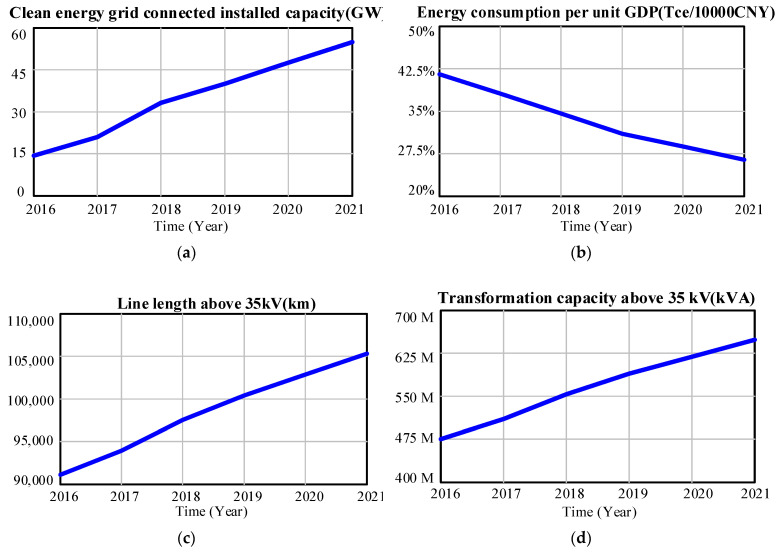

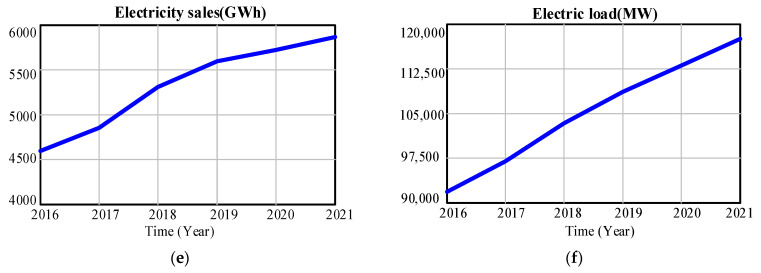
Simulation value of ESG investment state variable of grid company from 2016 to 2021, including (**a**) Clean energy grid-connected installed capacity; (**b**) Energy consumption per unit GDP(Tce/10000CNY); (**c**) Line length above 35 kV; (**d**) Transformation capacity above 35 kV; (**e**) Electricity sales; (**f**) Electric load.

Figure 7 shows the historical simulation results of clean energy grid-connected installed capacity, energy consumption per unit GDP, transformation capacity above 35 kV, line length above 35 kV, power load, and electricity sales in the Province from 2016 to 2021. Compare them with the actual values in historical years, and calculate the relative error between the simulated value and the actual value. When the relative error of each index is less than 10% each year and the average error over the years is less than 5%, the model is considered qualified.

#### 3.2.1. Model Validation of Environmental Investment Subsystem

Table 3 shows the comparison results between the simulation values and actual values of clean energy grid-connected installed capacity and energy consumption per unit GDP in the Province from 2016 to 2021. It can be seen from Table 3 that the relative errors of the two indicators in each year are less than 10%. After calculation, the average relative errors of the two indicators are 4.23% and 4.21% respectively, less than 5%, which is within the acceptable range. Therefore, the environmental investment SD simulation model constructed is reliable.

#### 3.2.2. Model Validation of Social Investment Subsystem

Table 4 shows the comparison between the simulated and actual values of the line length above 35 kV and the transformation capacity above 35 kV in the Province from 2016 to 2021. According to Table 5, the relative errors of the two indicators are less than 5%. After calculation, the average relative errors over the years are 0.82% and 1.92% respectively, which are acceptable.

#### 3.2.3. Model Validation of Governance Investment Subsystem

Table 5 shows the comparison between the simulated and actual values of electric load and electricity sales in the Province from 2016 to 2021. According to Table 6, the relative error degree of power load and electricity sales simulation is less than 10%. Through calculation, the average relative errors over the years are 1.55% and 2.33% respectively. Within an acceptable reasonable range, the SD simulation model of governance benefits built is reliable.

### 3.3. Model Prediction

#### 3.3.1. Model Prediction of Environmental Investment Subsystem

**Figure 8:**
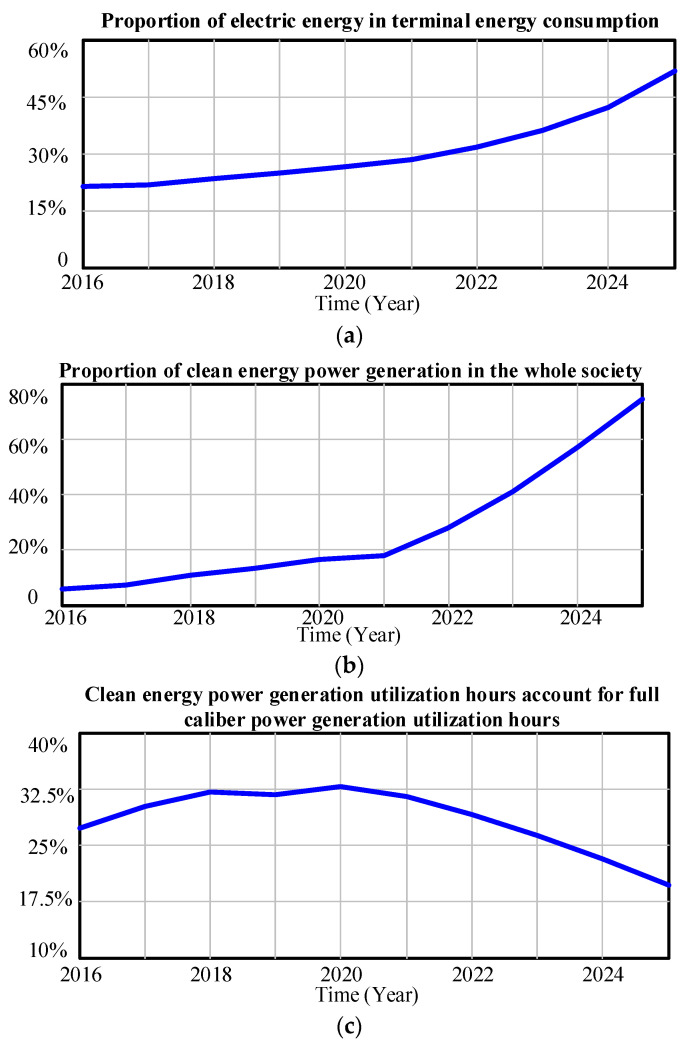
(**a**) The predicted value of the proportion of electric energy in terminal energy consumption from 2016 to 2025; (**b**) Predicted value of the proportion of clean energy power generation in the whole society from 2016 to 2025; (**c**) The predicted value of clean energy power generation utilization hours account for full caliber power generation utilization hours from 2016 to 2025.

The environmental investment benefit of the grid company is mainly characterized by the proportion of electric energy in terminal energy consumption, the proportion of clean energy power generation in the whole society, and the proportion of clean energy power generation utilization hours in full caliber power generation utilization hours. The simulation results of relevant indicators are shown in Figure 8.

It can be seen from Figure 8 that between 2016 and 2025, the proportion of electric energy in terminal energy consumption in the Province and the proportion of clean energy power generation in the whole society have maintained a high proportion of growth every year. In recent years, the development of clean energy has been a global theme. China has promoted many measures such as “double carbon” in recent years, and vigorously promoted the development of new energy. In this context, the proportion of clean energy power generation and the proportion of electric energy in terminal energy consumption will continue to increase rapidly in the future. In addition, the proportion of clean energy power generation utilization hours in full caliber power generation utilization hours in the Province from 2021–2025 will gradually decrease from 2022 to 2025, and the decrease rate will be rapid. The reason may be that wind power, photovoltaic, and other new energies are greatly affected by the time characteristics of resources, and the actual utilization efficiency is low. Faced with this situation, the Province should continue to promote technological progress, improve the performance and economy of new energy power generation, and then improve the efficiency of clean energy utilization.

#### 3.3.2. Model Prediction of Social Investment Subsystem

**Figure 9:**
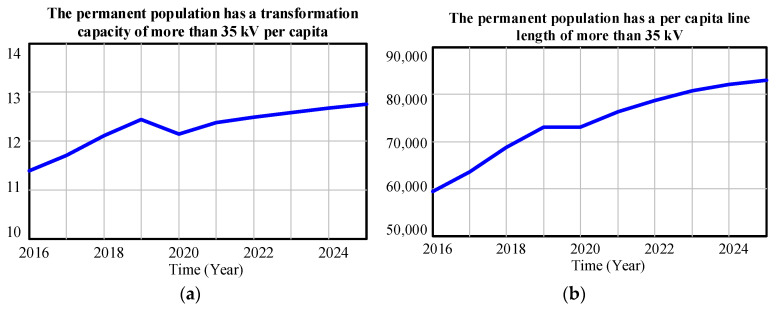
(**a**) The permanent population has a transformation capacity of more than 35 kV per capita from 2016 to 2025; (**b**) the Predicted value of the proportion of the permanent population has a per capita line length of more than 35 kV from 2016 to 2025.

Figure 9 shows the simulation prediction results of the line length above 35 kV per capita of the permanent population and the transformer capacity above 35 kV per capita of the Province from 2016 to 2025. The two indicators show a steady upward trend in 2022–2025. The growth of per capita line length and variable capacitance shows that the investment and construction of the Province in its power grid has to some extent met the increase of power consumption demand for production and residents’ lives in the Province, which has played a certain role in driving the development of the region. On the other hand, from the perspective of growth rate, both of them are slowing down gradually. In the context of rapid population growth, the Province should continue to strengthen the construction of energy infrastructure to improve the reliability of energy system supply and security assurance capability in order to avoid the potential shortage of power supply in the future.

#### 3.3.3. Model Prediction of Social Investment Subsystem

**Figure 10:**
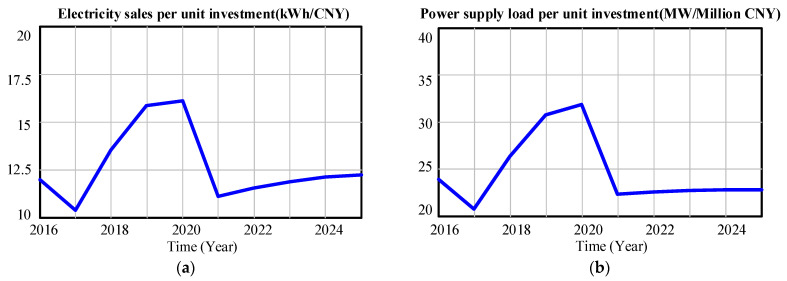
(**a**) The predicted value of the proportion of electricity sales per unit investment from 2016 to 2025; (**b**) the Predicted value of power supply load per unit investment from 2016 to 2025.

Figure 10 shows the simulation prediction results of power supply load per unit asset and electricity sales per unit asset in the Province from 2016 to 2025. It can be seen from Figure 8 that, benefiting from the rapid growth of the power supply load of the province from 2018 to 2021, the unit investment in electricity sales will reach 16.1091 kWh/CNY in 2020, and the unit investment in power supply load will reach 318.227 MW/million CNY in 2020. However, in 2021, both the unit investment power supply load and the unit investment electricity sales in the Province have decreased significantly, and the unit investment power supply load and the unit investment electricity sales have been at a low level from 2022 to 2025. The reason why the unit investment in power supply load and electricity sales in The Province has decreased year by year may be that the expected grid investment scale will grow rapidly from 2022 to 2025, and the growth rate of load and electricity sales will be lower than the growth rate of total grid assets in the Province. In order to effectively improve the unit investment power supply load and unit investment electricity sales of the power grid, the power grid company should pay attention to improving the level of project investment management. For regions with high expected power load growth and weak internal network structure of the power grid, it is necessary to reasonably increase the power grid investment in this region to give full play to the power grid investment benefits. For regions with low expected power load growth and strong internal network structure of the power grid, the power grid investment in the region should be reasonably reduced.

#### 3.3.4. Prediction of Investment Scale and Investment Weight

Based on the above analysis, it can be seen that under the given investment scale, the social benefits of the grid company meet the expectations. However, in terms of environmental investment, from 2022 to 2025, the proportion of clean energy power generation utilization hours to full caliber power generation utilization hours in the Province is expected to decline. In terms of governance investment, it is expected that the unit investment power supply load and unit investment electricity sales in the Province will be at a low level from 2022–2025. In order to effectively improve the investment environment and governance benefits of the Power Grid Company, it is set that the utilization hours of clean energy power generation in the Power Grid Company will account for 30% of the utilization hours of full caliber power generation in 2025, and the unit investment electricity sales will reach 17 kWh/CNY in 2025, The unit investment in power supply has a load of 320 MW/million CNY. Based on this, the proportion of the expected ESG investment scale of the power grid company from 2022 to 2025 is revised. The revised investment results of the Power Grid Company from 2022 to 2025 are shown in Table 6.

## 4. Conclusions

This paper applies the SD theory to the ESG investment of China Power Grid Corporation, selects the key indicators that reflect the ESG investment benefits of power grid companies from the three aspects of ESG, and constructs the SD model of the ESG investment of power grid companies by analyzing the causal relationship between the investment of power grid companies and the indicator variables. Then, taking a provincial Power Grid Company in China as the object, the process of ESG investment scale allocation of the power grid company is simulated by SD using Vensim PLE software. The model simulation takes the ESG investment scale as the input, and the change value of each index with the year as the output, which clarifies how much investment income will be brought by a certain ESG investment scale of the Power Grid Corporation. Through the mapping relationship between key indicators and investment amount, it effectively reflects the actual input-output efficiency of the ESG investment of power grid companies and analyzes the defects of the current ESG investment of power grid companies. The mathematical relationships between each variable cannot always be described by very precise formulas, influenced by the modeler’s knowledge, leading to the problem of low accuracy of the final simulation prediction results. Compared with the traditional static analysis method, the SD model can analyze the ESG investment benefits of power enterprises on multiple time scales and guides the ESG investment scale and investment weight of the power grid companies in the next few years.

## Figures and Tables

**Table 1:** Initial value setting of model variables.

Variable Name	Initial Value	Unit
Total assets	2803	million CNY
Investment in fixed assets	383.713	million CNY
GDP	76,086	million CNY
Permanent population	79,986	thousand person
Total electricity consumption	5458.95	GWh
Full caliber power generation utilization hours	4750	Hour
Electricity sales	4595.36	GWh
Power load	91,840	Mw
Length of lines above 35 kV	91,118.9	km
Transformation capacity above 35 kV	584.5	kVA
Marketable trading electricity	30.7261	GWh
Energy consumption per unit GDP	0.4147	Tce/million CNY
Clean energy grid-connected installed capacity	14,214.3	Mw

**Table 2:** Auxiliary variable table function formula setting.

Variable Name	Unit	Table Function Formula
Total assets	million CNY	“Total assets GRARF” ([(2016,0)–(2025,5000)], (2016,2803), (2017,2905), (2018,3025.54), (2019,3115.18), (2020,3200.04), (2021,3263.07), (2022,3458.78), (2023,3666.31), (2024,3886.29), (2025,4119.46))
Investment in fixed assets	million CNY	“Investment in fixed assets GRARF” ([(2016,0)–(2025,700)], (2016,383.713), (2017,466.911), (2018,392.221), (2019,353.33), (2020,355.326), (2021,527.44), (2022,549.487), (2023,572.456), (2024,596.384), (2025,621.313))
GDP	million CNY	“GDP GRARF” ([(2016,0)–(2025,200000)], (2016,76086), (2017,85901), (2018,92595), (2019,99632, (2020,102719), (2021,116364), (2022,122764), (2023,129516), (2024,136639), (2025,144155))
Total electricity consumption	GWh	“Total electricity consumption GRARF” ([(2016,0)–(2025,9000)], (2016,5458.95), (2017,5807.89), (2018,6128.27), (2019,6264.36), (2020,6373.71), (2021,7101.16), (2022,7420.72), (2023,7754.65), (2024,8103.61), (2025,8468.27))
Full caliber power generation utilization hours	hour	“Full caliber power generation utilization hours GRARF” ([(2016,3000)–(2025,6000)], (2016,4750), (2017,4478), (2018,4080), (2019,3849), (2020,3738), (2021,3991), (2022,4111), (2023,4234), (2024,4361), (2025,4492))
permanent residents	Ten thousand person	“permanent residents GRARF” ([(2016,0)–(2025,10000)], (2016,7998.6), (2017,8029.3), (2018,8050.7), (2019,8070.2), (2020,8474.8), (2021,8505.4), (2022,8760.56), (2023,9023.38), (2024,9294.08), (2025,9572.9))

**Table 3:** Comparison between simulation value and actual value of key indicators of environmental investment.

Year	Simulation Value of Clean Energy Grid Connected Installed Capacity (GW)	Actual Value of Clean Energy Grid Connected Installed Capacity (GW)	Relative Error	Simulation Value of Energy Consumption Level per Unit GDP (Tce/10,000 CNY)	Actual Value of Energy Consumption Level per Unit GDP (Tce/10,000 CNY)	Relative Error
2016	14.2143	14.2143	0.00%	0.4147	0.4147	0.00%
2017	21.5355	20.2745	6.22%	0.3829	0.3729	2.68%
2018	28.7693	28.9860	0.75%	0.3517	0.3492	0.72%
2019	36.0904	34.2832	5.27%	0.3183	0.3373	5.63%
2020	43.4088	40.4461	7.33%	0.2979	0.3286	9.34%
2021	50.7277	53.8647	5.82%	0.2764	0.2968	6.87%

**Table 4:** Comparison between simulation value and actual value of key indicators of social investment.

Year	Simulation Value of Line Length Above 35 kV (km)	Actual Value of Line Length above 35 kV (km)	Relative Error	Simulation Value of Transformation Capacity above 35 kV (kVA)	Actual Value of Transformation Capacity above 35 kV (kVA)	Relative Error
2016	91,118.87	91,118.87	0.00%	475,156,100	475,156,100	0.00%
2017	93,912.00	95,152.39	1.30%	510,291,000	529,710,730	3.67%
2018	97,524.90	97,837.59	0.32%	552,958,000	570,897,580	3.14%
2019	100,414.21	101,295.16	0.87%	589,406,000	603,288,330	2.30%
2020	102,840.44	102,681.73	0.15%	618,843,000	624,045,630	0.83%
2021	105,291.71	107,710.28	2.25%	648,703,000	659,160,680	1.59%

**Table 5:** Comparison between simulation value and actual value of key indicators of governance investment.

Year	Simulation Value of Electric Load (MW)	Actual Value of Electric Load (MW)	Relative Error	Simulation Value of Electricity Sales (GWh)	Actual Value of Electricity Sold (GWh)	Relative Error
2016	91,840	91,840	0.00%	4595.36	4595.36	0.00%
2017	96,996.2	102,140	5.04%	4850.35	4930.59	1.63%
2018	103,340	102,460	0.86%	5309.50	5297.02	0.24%
2019	108,678	107,590	1.01%	5594.80	5421.13	3.20%
2020	113,074	113,820	0.66%	5723.99	5528.73	3.53%
2021	117,526	119,590	1.73%	5862.28	6193.54	5.35%

**Table 6:** 2022–2025 Selected provincial Power Grid Company Investment Scale Guidance.

Year	Total Assets (Million CNY)	Investment in Fixed Assets (Million CNY)	Proportion of Environmental Investment	Proportion of Social Investment	Proportion of Governance Investment
2022	3459	549.5	20%	42%	38%
2023	3671	573.5	21%	41%	38%
2024	3896	599.6	21%	40%	39%
2025	4129	622.3	23%	38%	39%

## Data Availability

Data available on request from the authors.
